# Analysis of comprehensive genomic profiling of solid tumors with a novel assay for broad analysis in clinical diagnostics

**DOI:** 10.1002/1878-0261.13812

**Published:** 2025-01-31

**Authors:** Guy Froyen, Pieter‐Jan Volders, Ellen Geerdens, Severine Berden, Joni Van der Meulen, Aaron De Cock, Stefanie Vermeire, Jacques Van Huysse, Marie de Barsy, Gabriela Beniuga, Wendy W. J. de Leng, Anne M. L. Jansen, Imke Demers, Zeliha Ozgur, Hendrikus Jan Dubbink, Ernst‐Jan M. Speel, Wilfred F. J. van IJcken, Brigitte Maes

**Affiliations:** ^1^ Laboratory for Molecular Diagnostics, Department of Clinical Biology Jessa Hospital Hasselt Belgium; ^2^ Faculty of Medicine and Life Sciences University of Hasselt Belgium; ^3^ Department Jessa & Science LCRC (‐MHU) Hasselt Belgium; ^4^ Department of Biomolecular Medicine Ghent University Ghent Belgium; ^5^ Molecular Diagnostics Ghent University Hospital (MDG) Ghent University Hospital Ghent Belgium; ^6^ Cancer Research Institute Ghent (CRIG) Ghent University Ghent Belgium; ^7^ Department of Pathology AZ Sint‐Jan Brugge AV Bruges Belgium; ^8^ Institute of Pathology and Genetics (IPG) Gosselies Belgium; ^9^ Department of Pathology University Medical Centre Utrecht The Netherlands; ^10^ Department of Pathology Maastricht University Medical Center Maastricht The Netherlands; ^11^ Genomics Core Facility Erasmus University Medical Center Rotterdam The Netherlands; ^12^ Department of Pathology Erasmus MC Cancer Institute Rotterdam The Netherlands

**Keywords:** comprehensive genomic profiling (CGP), diagnostic assay validation, next‐generation sequencing (NGS), solid tumors

## Abstract

Somatic multigene analysis by next‐generation sequencing (NGS) is routinely integrated in medical oncology for clinical decision‐making. However, with the fast‐growing number of recommended and required genes as well as pan‐cancer biomarkers, small panels have become vastly insufficient. Comprehensive genomic profiling (CGP) is, thus, required to screen for clinically relevant markers. In this multicentric study, we report on an extensive analysis across seven centers comparing the results of the novel OncoDEEP CGP assay with the diagnostically validated TruSight Oncology 500 (TSO500) kit on 250 samples. Overall concordance was 90% for clinically relevant gene variants and >96% for more complex biomarkers. Agreement for fusion detection was 94% for the 11 overlapping clinically actionable driver genes. The higher coverage uniformity of OncoDEEP compared to TSO500 allows users to pool more samples per sequencing run. Tertiary data analysis, including reporting, is integrated in the OncoDEEP solution, whereas this is an add‐on for TSO500. Finally, we showed that, analytically, the OncoDEEP panel performs well, thereby advocating its use for CGP of solid tumors in diagnostic laboratories, providing an all‐in‐one solution for optimal patient management.

## Graphical abstract

In molecular cancer diagnostics, comprehensive genomic profiling (CGP) is going to replace the small NGS panels since it provides all clinically relevant somatic variants as well as genomic biomarkers with clinical value. Here, we compared two CGP assays and demonstrate that the choice for diagnostic implementation will depend on the specific requirements of the laboratory because each assay has its pros and cons.

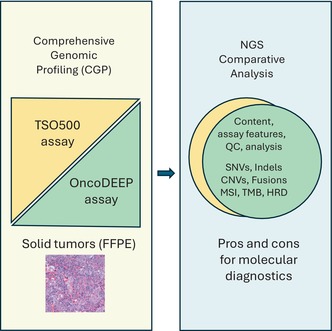

**Abbreviations**
C: center
CGP: comprehensive genomic profiling
CN: copy number
CNV: copy number variation
FC: fold change
FFPE: formalin‐fixed, paraffin‐embedded
GIS: genomic instability score
HRD: homologous recombination deficiency
indels: subtle insertions and deletions
LoD: limit of detection
MSI: microsatellite instability
MSS: microsatellite stable
NGS: next‐generation sequencing
SNV: single nucleotide variant
TMB: tumor mutation burden
UMI: unique molecular identifier
VAF: variant allele frequency

## 1 Introduction

Major advances in the insights of the pathophysiological characteristics of cancer resulted in a growing number of clinical targets that are exploited for the development of novel therapies. Recently, a large number of innovative cancer therapies have been implemented in clinical practice, often based on targeted treatment [1]. Moreover, the number of approvals of genomic biomarker‐driven cancer drugs by the Food and Drug Administration (FDA) and the European Medicines Agency (EMA) has grown rapidly [2], and the many ongoing clinical trials are expected to result in several more targeted therapies in the coming years. The major challenge to exploit these targeted treatments, however, is the ability to detect the corresponding clinical targets at the molecular level. Ideally, the different oncogenic mutation types including single nucleotide variants (SNVs), subtle insertions and deletions (indels), copy number variations (CNVs), gene fusions, and splice variants of all cancer‐related genes should be detected reliably and simultaneously. A very important additional advantage of comprehensive genomic profiling (CGP) panels is the ability to detect pan‐tumor markers including microsatellite instability (MSI), tumor mutation burden (TMB), and homologous recombination deficiency (HRD). In conjunction with histopathological findings, somatic variants can provide useful information for diagnosis as well as for risk stratification, prognosis, therapeutic response, and prognostication of resistance in multiple tumor types [3, 4]. Moreover, it allows the enrollment in clinical trials and can assist in potential off‐label treatment recommendations that can benefit patients. However, most molecular diagnostic laboratories still analyze smaller next‐generation sequencing (NGS) gene panels because of its acceptable cost in relation to the local reimbursement system and its lower bioinformatic burden. On the other hand, the use of comprehensive panels can make optimal use of the scarcely obtained tumor material and allows to screen multiple parameters simultaneously. Therefore, CGP is more and more emerging as the technology of choice in clinical practice. To date, large panels have shown their clinical utility in several tumor types [5, 6, 7]. Finally, germline variants that confer cancer predisposition can also be detected allowing constitutional follow‐up. Although the simultaneous analysis of all clinically relevant information confers major time and economic profit, it requires a much more challenging validation plan that has to include extensive bioinformatic analysis. For that reason, CGP assays are rarely custom‐designed. So far, only a few commercial CGP panels have been validated in diagnostic laboratories including the FoundationONE CDx (F1CDx, 324 genes; FoundationMedicine) [8], the TruSight Oncology 500 (TSO500, 523 genes; Illumina) [9, 10], and the Oncomine Comprehensive assay v3 (OCAv3, 501 genes; ThermoFisher) [11]. Clinically useful CGP assays have significantly evolved during the past years allowing reliable variant and biomarker detection at a reasonable cost, making CGP soon the standard of care in clinical oncology [7].

In this multicentric study, we describe the comparison of the performance of the novel OncoDEEP CGP assay with the validated TSO500 assay using 234 diagnostic and 8 reference samples. In addition, the successful analytical validation of the OncoDEEP assay is presented.

## 2 Materials and methods

### 2.1 Study setup

Comparative analysis of the OncoDEEP assay was performed in the diagnostic laboratories of seven centers of which four (Jessa Hospital Hasselt, University Hospital Ghent, General Hospital St‐Jan Bruges, and the Institute of Pathology and Genetics Gosselies) are located in Belgium and three (University Medical Centre Utrecht, Maastricht University Medical Center, and Erasmus MC Cancer Institute Rotterdam) in The Netherlands (Table 1). Three centers (C2, C3, and C4) perform NGS analysis in routine cancer diagnostics using accredited small gene panels and have expertise with TSO500 analysis in the clinical BALLETT study (NCT05058937). Four centers (C1, C5, C6, and C7) offer accredited small gene panels as well as TSO500 screening in routine. This study was carried out in compliance with the principles outlined in the Declaration of Helsinki. Because these analyses had been done on residual sample material, no formal ethical approval is required. According to the Belgian law of 19 December 2008 with number N. [2008‐4682 C‐2008/18385], no written informed consent is required either. All tests were performed in accordance with relevant guidelines and regulations in Belgium and The Netherlands.

**Table 1:** Overview of the seven centers (C1–C7), the number of samples processed for each variant type, and the sequencing information for the comparative analysis of the TSO500 and OncoDEEP CGP assays. #, number of; bp, base pairs; na, not applicable; nd, not determined.

Center	C1	C2	C3	C4	C5	C6	C7	Total
# diagnostic DNA samples	75	21	25	27	8	51	27	234
DNA extraction method	Maxwell FFPE	QIAamp FFPE	Magcore	Qiacube	Chelex/Maxwell	Maxwell FFPE	Maxwell FFPE	
# reference DNA samples	2	1	6	2	0	0	0	11
Based on TSO500 results								
# SNVs and indels	232	77	71	80	18	118	78	674
# amplifications (FC ≥6)	12	1	6	11	nd	nd	1	31
# MSI‐high (>20%)	7	1	1	0	nd	nd	1	9
# TMB‐high (>16)	10	4	3	1	2	nd	2	22
# HRD pos (GIS ≥42)	nd	nd	0	nd	nd	nd	nd	0
# diagnostic samples with rearrangements	11	28	3	2	0	0	23	67
RNA extraction method	Maxwell FFPE	Maxwell FFPE	Magcore	Maxwell FFPE	na	na	Maxwell FFPE	
# reference RNA samples	2	2	2	2	0	0	0	8
Based on TSO500 results								
# gene fusions	34	58	29	28	0	0	23	172
# splice variants (AR, EGFR, MET)	7	9	2	2	0	0	0	20
Sequencer TSO500 samples	NextSeq500	NovaSeq6000	NextSeq550Dx	NextSeq500	NovaSeq6000	NextSeq500	NextSeq550	
Flowcell TSO500 samples	HO v2.5	SP	HO v2.5	HO v2.5	SP	HO v2.5	HO v2.5	
# TSO500 samples pooled	8	16	8	8	16	8	8	
Sequencer OncoDEEP samples	NextSeq500	NextSeq2000	Nextseq550Dx	NextSeq500	NextSeq500	NextSeq500	NextSeq500	
Flowcell OncoDEEP samples	HO v2.5	P1	HO v2.5	HO v2.5	HO v2.5	HO v2.5	HO v2.5	
# OncoDEEP samples pooled	24	8	24	16	24	24	24	

### 2.2 Sample selection

All samples used for the OncoDEEP analysis had been analyzed previously with TSO500 in the seven centers and were selected from samples collected from October 2020 to July 2023. The number of samples selected by each institute is provided in Table 1. A total of 234 diagnostic DNA samples were analyzed for SNVs, indels, and amplifications. On many of those the pan‐cancer biomarkers MSI and TMB could also be compared. Exon skipping and fusion detection on RNA was performed on 175 samples, including the references. Seventy diagnostic samples harbored a known rearrangement based on the TSO500 analyses. Tumor content (TC) of the selected tumor regions ranged from 15% to 90% (mean 59.2; SD 21.5). Additionally, four centers analyzed one to six reference DNA (HD753 and HD827, HorizonDx, Cambridge, UK; four SeraSeq FFPE HRD, SeraCare, Milford, MA, USA) and two reference RNA (SeraSeq FFPE fusion RNA mix v4 and SeraSeq NTRK fusion RNA, SeraCare) samples. DNA and RNA from routine clinical samples, extracted from formalin‐fixed paraffin‐embedded (FFPE) tumor tissue and stored for a maximum of 1.5 y at −20 °C and −80 °C, respectively, were used for OncoDEEP analysis. Since all samples had been previously analyzed with the TSO500 kit both assays thus started from exactly the same material except for center C5 that had to re‐extract DNA because Chelex‐extracted DNA does not work in the OncoDEEP assay. Extraction methods for each center are listed in Table 1. The selected samples comprised a representation of many different tumor types (Table S1). Based on the TSO500 data, the different genomic alterations from these samples could be used for a thorough comparison with OncoDEEP analysis, including 674 SNVs and indels, 33 amplifications with a fold change (FC) ≥6, 172 fusions, and 20 splice variants. In pan‐cancer biomarker analysis, nine samples were MSI‐high (MSI‐H; ≥20%) and 22 were TMB‐high (TMB‐H; ≥16 mut·Mb^−1^). For HRD analysis, we could only include eight diagnostic samples, which were all HRD negative (Genomic Instability Score, GIS <42 and no pathogenic BRCA1/2 variant), and four SeraCare HRD reference samples (SeraSeq). Molecular alterations present in the DNA and RNA reference materials can be found at the company's websites (https://horizondiscovery.com/en/reference‐standards and https://www.seracare.com/).

### 2.3 Content of the CGP panels

The OncoDEEP panel is a hybrid‐capture‐based panel with a total panel size of 1.8 Mb. The panel covers 638 cancer‐related genes allowing the detection of SNVs, indels, CNVs, and LOH at DNA level. Information on the genomic biomarkers TMB, MSI, and HRD is standardly provided as well. Clinically important rearrangements at the RNA level can be detected in 11 genes (13 in current panel) and splice variants in nine genes. Differences in content with the TSO500 panel are shown in Table 2. The comparative evaluation of both CGP assays can be conducted for the overlapping genes only, that is, SNVs and indels in 516 genes, amplifications in 59 genes, fusions in 11 genes, and exon skipping events in three genes. The gene content of each panel for each those is provided in Table S2. For the biomarkers MSI, TMB, and HRD, the status (e.g., High, Pos) as well as the observed measure (percentage, number, score) were also compared.

**Table 2:** Comparison of the gene content and features of the TSO500 and OncoDEEP panels. Future improvements are provided in between brackets.

Detection at DNA level		
	TSO500	OncoDEEP
Total size	1.9 Mb	1.8 Mb
	# genes	
SNVs and indels	523	638
CNV	59 (514^a^)	614
LOH	0 (514^a^, ^b^)	41
	Pan‐tumor biomarkers	
MSI	Yes	Yes
TMB	Yes	Yes
HRD	Yes^b^	Yes

Detection at RNA level		
	TSO500	OncoDEEP
	# driver genes	
Fusions	55	11 (13^c^)
Splice variants	3	9

a In current kit using DRAGEN analysis.

b In current kit as an add‐on to the assay.

c In current kit.

### 2.4 Library preparation and sequencing

Sequencing libraries of the OncoDEEP assay were generated in accordance with the protocol provided by the manufacturer. In short, up to 100 ng of DNA was enzymatically fragmented followed by end‐repair and A‐tailing. The DNA fragments were then ligated with adaptors and subjected to eight PCR cycles. After cleanup, up to eight pooled libraries were hybridized for 16 h with biotinylated oligonucleotide DNA baits enriched on streptavidin‐conjugated magnetic beads and subjected to a second PCR and cleanup step. For fusion genes and altered splicing detection, up to 200 ng of dried RNA was used for cDNA synthesis, which was then further processed as described for the fragmented DNA samples. All libraries were qualitatively checked on a BioAnalyzer (Agilent, Santa Clara, CA, USA) or Tapestation (Agilent) and quantified with a Qubit v3.0 fluorometer (Life Technologies, Gaithersburg, MD, USA). For sequencing, 16 or 24 libraries (DNA and RNA) were equimolarly pooled and 2 × 74 bp paired‐end sequenced on a High Output flow cell on a NextSeq500/550 instrument (Illumina, San Diego, CA, USA) in all but one center (Table 1). The library preparation and sequencing of the TSO500 assay have been described previously [9]. For those, eight pooled libraries (DNA and RNA) were 2 × 101 bp paired‐end sequenced on a High Output flow cell on a NextSeq500/550 in five centers while the NovaSeq6000 (Illumina) was used in two centers with 16 or 32 HT‐TSO500 libraries on a SP flow cell.

### 2.5 Data analysis

Run quality assessment was performed using the Sequence Analysis Viewer (SAV; Illumina). For OncoDEEP data analysis, the demultiplexed Fastq files automatically generated by the Local Run Manager (LRM; Illumina) software of the instrument were uploaded in the cloud‐based OncoKDM platform (OncoDNA) for downstream identification of genomic variants and pan‐cancer biomarkers. This tool also provides the clinical interpretation of the (likely) pathogenic variants and biomarkers. Data analysis of the TSO500 samples was done with the TSO500 Local App v2.0 (Illumina) as described previously [9], except for Center 6 that used Franklin (Genoox). HRD analysis was performed on the Dragen server (Illumina). Thresholds for the minimum coverage at each variant position were set at 150× for TSO500 and 80× for OncoDEEP as recommended by the providers. The VAF threshold for SNVs and indels was set at 5% for both assays. For the concordance evaluation, TSO500 variants with a coverage or VAF below the thresholds were not taken into account and amplifications with FC ≥ 6 were included. Variant classification was performed manually and based on the ComPerMed guidelines as described earlier [12].

All CGP data of the samples for both assays were entered by all centers in a template file to allow script‐based comparative analyses. The statistical analysis was performed using the R programming language (v4.3.0) and the R studio software. Descriptive statistics were utilized to summarize the data. The association between the status of TMB and MSI scores was evaluated using appropriate statistical tools including *t*‐tests, Fisher's exact test, and ANOVA. The Pearson correlation coefficient was used to assess the linear relationship between the allele ratios of the variants.

### 2.6 Analytical validation of the OncoDEEP assay

The analytical performance of the OncoDEEP assay was evaluated in a single center (C1) on 87 samples consisting of 79 retrospective clinical DNA and/or RNA samples representing more than 20 tumor types with a mixture of variants (SNVs, indels, CNVs, fusions, and exon skipping events) as well as with clinically relevant pan‐cancer biomarkers (MSI, TMB, and HRD). In addition, two DNA (HD827 and HD753) and two RNA (RNA fusion mix v4 and NTRK fusion RNA) reference samples carrying multiple pathogenic variants were included. Subsets of all samples were used to assess the precision, analytical sensitivity and specificity, limit‐of‐detection, and accuracy in a similar way as described previously for the TSO500 assay [9]. An overview of the number of samples and variants used to assess the different performance characteristics is shown in Table 3.

**Table 3:** Overview of the number of variants and biomarkers used to assess each performance characteristic for the analytical validation of the OncoDEEP assay. The number of unique samples (# smpls) for each characteristic is indicated as well. #, number of; amplif, amplifications; ex, exon; smpls, samples; D + R, DNA + RNA.

Performance characteristic	# of variants/pos biomarkers at DNA level						DNA	# of variants at RNA level		RNA	Total (D + R)
	SNV	Indel	Amplif	MSI‐H	TMB‐H	HRD	# smpls	Ex skipping	Gene fusion	# smpls	# smpls
Precision	18	8	1	2	2	0	4	2	26	2	6
Sensitivity/specificity	>150	>80	24	6	17	12	79	3	31	79	83
Limit‐of‐Detection	4	10	2	0	0	0	4	2	26	2	6
Accuracy	>150	>80	24	6	17	12	86	3	31	86	87

## 3 Results

### 3.1 TSO500 versus OncoDEEP assay

Table 4 summarizes the main differences between both assays in input amount, library preparation, and sequencing. In comparison with the TSO500, the OncoDEEP assay does not need an ultrasound sonicator since DNA fragmentation is performed enzymatically. OncoDEEP requires the drying of the RNA in a speedvac dryer, which allows low‐concentrated samples to be used, but also negatively affects the RNA quality in one center. Further, OncoDEEP does not make use of unique molecular identifiers (UMIs) to reduce the PCR‐induced error rate while TSO500 does. Up to eight libraries can be pooled in the single overnight hybridization capture step in the OncoDEEP assay. In the TSO500 protocol, next to the overnight hybridization, each library requires one additional short hybridization step to improve the on‐target capturing. Normalization of the libraries is bead‐based in TSO500 and quantitation‐based in OncoDEEP. In our hands, bead‐based normalization provides a more uniform pooling of the libraries. For sequencing, the number of pooled OncoDEEP libraries is two to three times higher when compared to TSO500 libraries. Finally, tertiary analysis (OncoKDM) is standardly integrated in the OncoDEEP assay while the Illumina connected insights (ICI) tool is an add‐on to the TSO500 kit.

**Table 4:** Summary of the main differences between the TSO500 and OncoDEEP assays at the time of usage. *Italic text* in brackets indicate the changes in the current protocols. #, number of; ICI, Illumina Connected Insights; UMI, Unique Molecular Identifier. Italic text between brackets indicate the current changes.

	TSO500 (Illumina)	OncoDEEP (OncoDNA)
	Pre‐analytics	
Recommended input	DNA: 40 ng	DNA: 100 ng *(40 ng)*
	RNA: 40 ng	RNA: 200 ng dried *(80 ng)*
	Library prep	
DNA Fragmentation method	Shearing	Enzymatic
Use of UMIs	Yes	No
Normalization	With beads	Quantification and dilution
	Hybridization capture	
Pooling before hyb	No	Yes (8 samples)
# Hybridization steps	2	1
	Sequencing on a NextSeq550	
Read length	2 × 101 bp	2 × 74 bp
#Samples per run	8; DNA + RNA	24; DNA + RNA
Flowcell NextSeq550Dx	HO v2.5–300 cycles	HO v2.5–150 cycles
	Data analysis	
Secondary analysis	TSO500 local app *(DRAGEN)*	OncoKDM
Tertiary analysis	*(ICI as an add‐on)*	OncoKDM
	Hands‐on time	
Hands‐on time	5 h	4 h

**Fig. 1:**
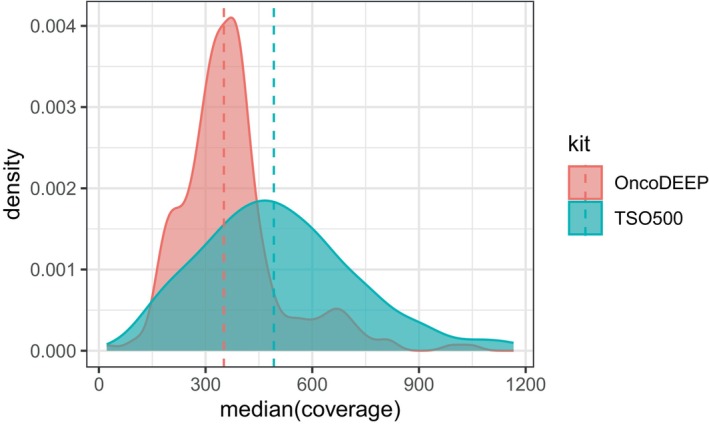
Distribution of the median coverage obtained with OncoDEEP (red) and TSO500 (blue). Data are extracted from NextSeq500 runs with libraries from 24 DNA + RNA OncoDEEP samples on a High Output (HO) v2.5 150 cycles flow cell, or 8 DNA + RNA TSO500 samples on a HO v2.5 300 cycles flow cell. The vertical dashed lines represent the mean coverage of all samples.

The sequencing performance metrics of the OncoDEEP runs in this study (mean % ≥ Q30: 92.3/mm^2^ and mean cluster pass filter: 87.3%) were very similar to those of TSO500 (92.0% and 89.0%, respectively). The median coverages of all samples for OncoDEEP are more uniform (369 ± 145) than those obtained for TSO500 (504 ± 217) (Fig. 1). As a consequence, more libraries can be pooled for sequencing on the same instrument.

### 3.2 Detection of SNVs and indels

**Fig. 2:**
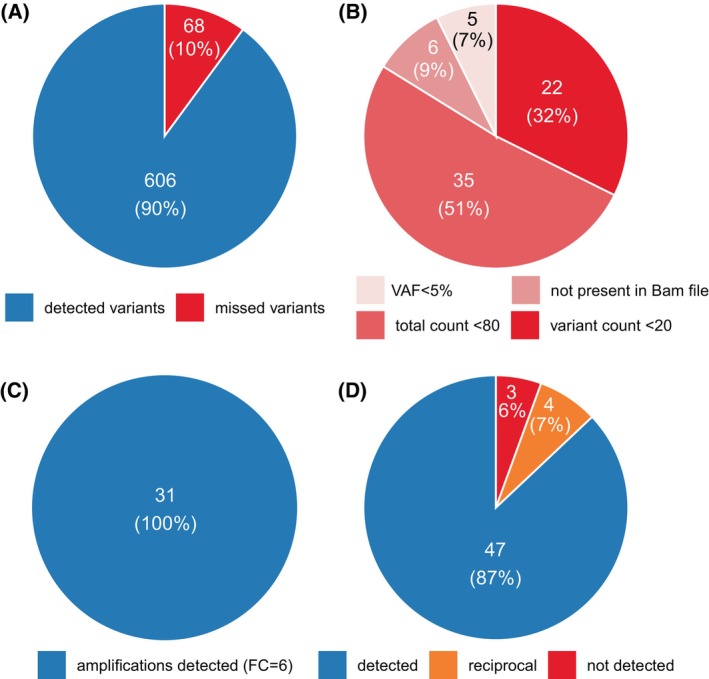
TSO500 variants not detected by OncoDEEP. (A) Of the 674 TSO500 (likely) pathogenic single nucleotide variants (SNVs) and insertion/deletions variants (indels), 90% were also detected by OncoDEEP. (B) Breakup of the missed variants (*n* = 68) in the OncoDEEP assay according to the cause. The majority (*n* = 57) were missed due to an insufficient number of reads at that position. (C) Percentage of gene amplifications with fold change (FC) ≥6 found with TSO500 that were also detected with the OncoDEEP assay. (D) Breakup of gene fusions and exon skipping events that were detected or not with OncoDEEP. Four (7%) were present as the reciprocal fusion.

For the evaluation of the detection of the TSO500‐derived pathogenic and likely pathogenic SNVs and indels by the OncoDEEP assay, we examined their presence and VAFs in 234 diagnostic samples. Because TMB‐high samples (median mutations per Mb of 114) could easily harbor more than 50 (likely) pathogenic variants in TSO500, we restricted the analyses to the 10 most clinically relevant (actionable) variants of each sample. As a consequence, a total of 674 TSO500 variants were included. Of those, 606 (89.9%) were also found with OncoDEEP while 68 (10.1%) were not (Fig. 2A).

**Fig. 3:**
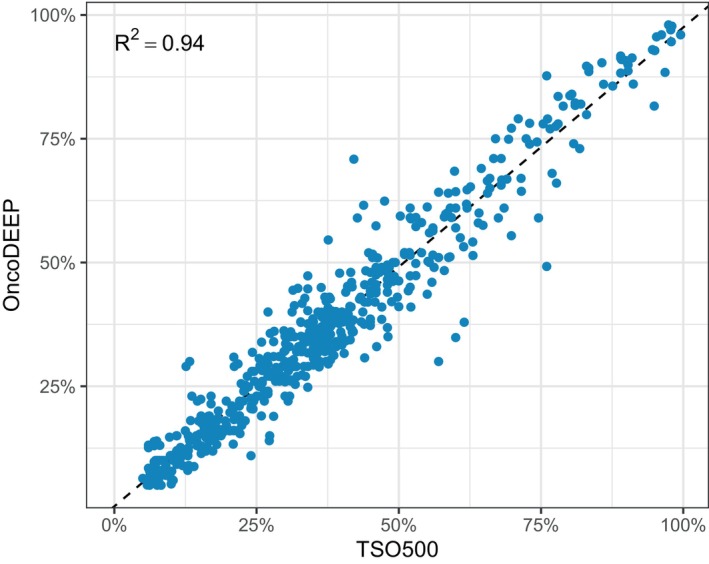
Comparison of the variant allele frequency (VAF) of variants detected with the TSO500 and OncoDEEP assays. Pearson correlation provides an *R* ^2^ of 0.94. The dotted line represents the linear regression.

The misclassification by the software of a variant as a VUS instead of a (likely) pathogenic variant (five variants) was not regarded as a missed variant. The 12 pathogenic and 56 likely pathogenic variants not detected with OncoDEEP are listed in Table S3. No skewing for the predominance of SNVs or indels was observed. Of the 68 missed variants, five variants had a VAF in TSO500 just above 5% and thus likely were excluded from the OncoKDM reports because of a VAF below the 5% threshold. For six TSO500 variants with VAF between 9.5% and 56%, no evidence whatsoever was available for those variants in the Bam file of the OncoDEEP analysis. The reasons for these discrepancies could not be retrieved. For the remaining 57 missed variants, the total number of reads at that position (35 variants) or the number of variant reads (22 variants) was below the OncoDEEP filter threshold of 80 and 20 reads, respectively (Fig. 2B). No correlation with TC was observed since the mean TC for samples carrying at least one missed variant (56%) was very similar to the mean TC of all analyzed samples (59%). Between the centers, however, the ratio of missed variants per sample varied from 0.13 to 0.65 suggesting center‐specific issues. Indeed, most of these variants were present in samples that did not pass the OncoKDM QC criteria, that is, mean coverage ≥250× or uniformity of coverage ≥90%, which vary significantly between centers too (Fig. S1). We could, however, not pinpoint whether these QC failures were related to the DNA/RNA quantity or quality scores. Importantly, the VAFs of the 606 variants detected with both assays were highly similar with a correlation coefficient (*R* ^2^) of 0.937 (Fig. 3).

Reference DNA samples HD753 and/or HD827 were analyzed in four centers (C1–C4). Of the 10 reported variants in HD753 and the 15 variants in HD827, all SNVs and indels with VAF ≥5% were detected. The variants with VAF <5% were excluded from the reports because of the hard filter.

### 3.3 Detection of copy number gains

The TSO500 software used in this study provided information on the potential amplification of 59 genes while all 638 genes can be checked for gains and losses in OncoDEEP. OncoKDM only reports amplifications with a copy number ≥6, which were compared for the overlapping 59 genes. In the 183 diagnostic samples tested, 31 amplifications were detected in these 59 genes with a fold change (FC) ≥6 in TSO500 (Table S4). Since the mean FC values (17.8) obtained for the 31 amplified genes in TSO500 were close to the mean copy number changes (CNs) given in OncoKDM (19.5), we can assume very similar amplification value calculations. Therefore, we can conclude that all amplified genes detected with TSO500 were also found with OncoDEEP (CN ≥6) giving a detection rate of 100% (Fig. 2C). For 12 gene amplifications detected with OncoDEEP with a CN≥6, the FC was between 4 and 6 in the TSO500 analysis. The genes that were most often amplified in our cohort were *CCND1* (8), *RPS6KB1* (5), *MYC* (4), and *EGFR* (4). Highest FCs (>40) were found for *EGFR, MDM2*, and *ERBB2*.

### 3.4 Gene fusions and alternative splicing

Based on the TSO500 fusion results, we selected 67 diagnostic samples carrying a fusion gene and/or exon skipping event. These samples included 58 fusions and 11 alternative splicing events yielding a total of 69 rearrangements. Of those, 54 (43 fusions and 11 splicing) had driver genes present in the OncoDEEP panel (*ALK, BRAF*, *FGFR2*, *FGFR3*, *NRG1*, *NTRK1*, *NTRK3*, *RET*, and *ROS1*) meaning that for 15 fusions, the driver genes were not included in OncoDEEP and thus could not be included in this comparative analysis. OncoDEEP revealed the same rearrangement for 47 (36 fusions and 11 splicing events) of 54 (Fig. 2D; Table S5). For the seven discordant fusion genes, the reciprocal fusion was detected in three cases (e.g., *ALK::TIMP3* vs. *TIMP3::ALK*) and for one case the *MYO18A::ROS1* was reported in OncoDEEP while the *MYO18A* was fused to the *ROS1*‐flanking gene *GOPC* as detected by TSO500. However, this inconsistency likely is due to an annotation error since *GOPC* overlaps the *ROS1* gene in Hg19. For the final three fusions not detected with OncoDEEP (with *ALK*, *BRAF*, and *NTRK3* drivers), no explanation could be given for their absence, also in the Bam files. Notably, aberrations with the same drivers were detected in several other samples including the reference RNA samples. Obviously, missing these clinically relevant fusions would have a negative impact on clinical management of these patients. Overall, for 51 of the 54 rearrangements (94.4%), a fusion or exon skipping event was found by the OncoDEEP assay.

Analysis of both reference RNA samples was performed in four centers (C1–C4). All 16 rearrangements of the RNA fusion Mix v4 were detected. In the NTRK fusion RNA sample, the same 12 fusions with *NTRK* driver genes were found but from the four *ETV6::NTRK3* fusions that use alternative breakpoints, only one was present in OncoKDM. Analyses of both reference samples with TSO500 detected all rearrangements.

### 3.5 Analysis of tumor agnostic biomarkers

**Fig. 4:**
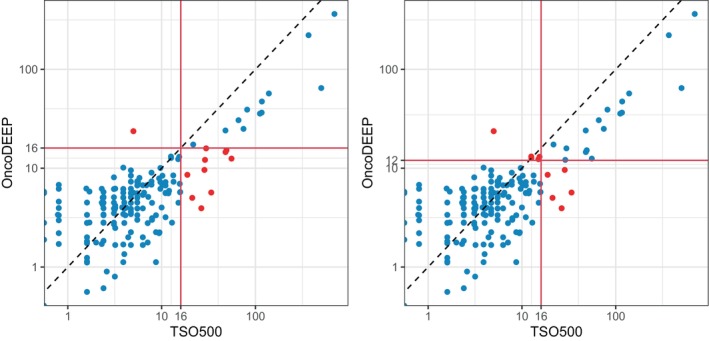
Comparison of tumor mutation burden (TMB) values (log‐scale) obtained with the TSO500 and OncoDEEP assays. Plots in which the OncoDEEP threshold was set at 16 mut·Mb^−1^ (A) or at 12 mut·Mb^−1^ (B). Red dots indicate the samples with discordant calls.

A total of 175 samples representing a variety of tumor types and mutational loads could be checked for their TMB values in both assays. Of those, 22 were found TMB‐H with TSO500 (≥16 mut·Mb^−1^). As OncoDEEP does not provide a threshold, using the same threshold revealed that nine of these 22 samples were TMB‐low while only a single sample had the opposite discordant call (Fig. 4A). Since TSO500 TMB values for these 175 samples were generally higher (mean 20 mut·Mb^−1^) than those obtained with OncoDEEP (mean 10 mut·Mb^−1^), decreasing the OncoDEEP threshold to 12 mut·Mb^−1^ resulted in the lowest number (9) of discordant calls. Moreover, TMB values were closer to the thresholds now (Fig. 4B). Using these thresholds, the overall agreement of TMB scoring was 94.9% (166/175). Values close to the threshold could deviate and thus should be interpreted with care.

**Fig. 5:**
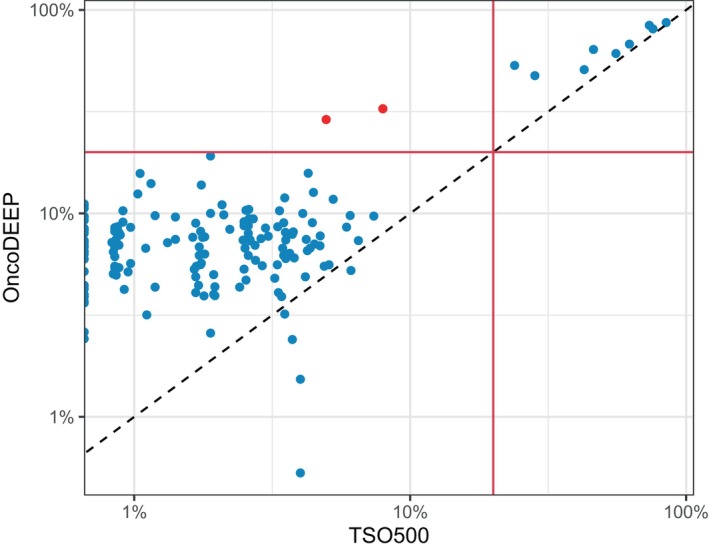
Microsatellite instability (MSI) ratio plot (log‐scale) for samples analyzed with TSO500 and OncoDEEP. Using a threshold of 20%, only two discordant calls (red dots) were present. The broken line indicates the theoretical perfect match.

In the 162 samples in which the MSI was calculated in both assays, the scoring of each sample as either microsatellite stable (MSS) or microsatellite instability high (MSI‐H) was evaluated. The MSI ratio is generally higher in OncoDEEP (median 6.9%) compared to TSO500 (median 1.8%). However, setting the threshold at 20% for both assays called all nine MSI‐H samples of TSO500 also MSI‐H with OncoDEEP. Only two additional MSI‐H samples were found with the OncoDEEP kit (Fig. 5). For one of both, immunohistochemistry was performed and showed an MSI‐H result based on loss of MSH2 and MSH6. In any case, the classification of samples as MSS or MSI‐H showed a high level of concordance with an overall agreement of 98.8% (160/162 cases).

Since the TSO500‐HRD panel was only recently introduced, no HRD data were available for the 234 diagnostic samples. An additional eight diagnostic samples were tested with both assays, but all were HRD negative. Therefore, we also tested the four SeraCare HRD reference samples, which revealed a GIS of 61 for the HRD very high, 53 for the HRD medium high, 29 for the HRD medium low, and 6 for the HRD low sample. Since the HRD threshold in OncoDEEP is ≥39, all four reference samples were correctly called. However, because of the low number of samples in this comparative analysis, no definitive conclusion can be made. We, therefore, tested additional samples in the validation study.

### 3.6 Validation of the OncoDEEP assay

The data of the multicentric comparative analysis of the OncoDEEP CGP assay prompted us to perform an analytical validation of this kit, conducted in a single center in a similar way as reported previously for TSO500 [9]. A total of 77 diagnostic and 2 reference DNA samples were included (Table 3) of which two failed QC. Both samples had a DNA input amount <20 ng, which was well below the recommended input of 100 ng. RNA analysis was performed on the same 77 diagnostic samples, which carried three fusion genes and one *MET* exon 14 skipping event. Additionally, both SeraCare reference RNA samples harboring 31 fusion events were also included in this validation.

The precision of the OncoDEEP assay was first tested on two diagnostic samples carrying four DNA variants (three SNVs and one indel) of which one was in the exon 14 splice site of *MET*. Both samples were tested in triplicate for repeatability (intra‐run) and reproducibility (inter‐run). The VAFs of the variants identified were highly similar in each of the tests and the *MET* exon 14 skipping variant was detected in all RNA assays as well. Additionally, both DNA and RNA reference samples were tested in duplicate in the same and/or in different runs. From the 25 variants (18 SNV and 7 indels) in the reference HDx samples, 21 variants with VAFs between 5% and 30% were consistently detected while four variants with VAFs between 1% and 4.4% were not, most likely due to the VAF threshold of 5%. The *MYC* amplification as well as MSI‐H and TMB‐H status were confirmed in both DNA reference samples. In the SeraCare RNA samples, the 26 fusions as well as both exon skipping events were consistently detected. Since OncoKDM does not provide the number of reads supporting the fusion, we cannot compare these numbers with those obtained by TSO500. In conclusion, the precision of the OncoDEEP assay was maximal since all clinically relevant variants, CNVs, fusions, and MSI and TMB values were reproducibly detected.

To assess the sensitivity and specificity at the DNA level, from the 262 SNV and indel variants that were detected by TSO500, 240 (91.6%) were also found with OncoDEEP using the standard filter settings in OncoKDM. Again, those that were absent were mostly due to a too low coverage at that variant position as described earlier. We did not, however, find any false positive variants with VAF ≥5%. From the 19 amplifications with FC ≥6 in TSO500, 18 (94.7%) were equally detected with OncoDEEP. One *MYCN* amplification was discordant. For MSI analysis, concordant data were present for 76 of 77 (98.7%) samples. TMB analysis was performed using the thresholds for TMB‐H at 16 and 12 for TSO500 and OncoDEEP, respectively. Consequently, 72 of 77 (93.5%) samples were concordant. For HRD analysis, an additional 10 samples were tested of which five were positive with TSO500 (GIS ≥42 and/or *BRCA1/2* mutation). These samples were also positive with OncoDEEP (GIS ≥39 and/or *BRCA1/2* mutation) but from the five TSO500‐HRD‐negative samples, two were also found positive in OncoDEEP both with a GIS of 41. The TSO500‐derived GIS for these samples was 15 and 23 (Table S6). Taken together, the sensitivity and specificity of the OncoDEEP kit for the pan‐cancer biomarkers MSI and HRD was >93%. At the RNA level, three of the four rearrangements were detected in the diagnostic validation samples. The apparently missed *FGFR1::SLIT1* fusion did not include the kinase domain of FGFR1 and therefore, the targeting probe to detect this fusion was not included in the OncoDEEP panel prohibiting its detection. All gene fusions of both reference RNA samples were successfully detected. Again, for the four available *ETV6::NTRK3* fusions, only one of those was in OncoKDM.

The limit‐of‐detection (LoD) analysis was performed by mixing samples at variable ratios to obtain contributions to the mixtures of 75%, 30%, and 10%. The VAFs of the 20 variants (9 SNVs and 11 indels) varied between 18% and 60% in the undiluted samples, and amplification of ERBB2 and KRAS had CNs of 26 and 28, respectively. As expected, gradual dilutions resulted in a linear decrease of the VAFs (Fig. S2) and FCs. Since the VAF reporting threshold was set at 5%, variants with VAF <5% were filtered out. As a consequence, the LoD of the variants was between 5% and 14% likely because the subsequent dilution resulted in a VAF <5%. Similarly, both *KRAS* and *ERBB2* amplifications were still detected in the 25% contribution analysis with CNs of 6 and 18, respectively, but not anymore in the 10% mixtures. Fusion detection for LoD analysis was tested on 3 and 10 times diluted RNA reference samples and revealed detection of all fusions as well as both exon skipping events in each dilution (data not shown). However, the *GOPC::ROS1* fusion was also present in each of the diluted reference samples while absent in the undiluted samples suggesting it to be a false positive read through transcript.

Finally, we checked the effect of the DNA and RNA input amount of the 77 diagnostic samples on the success rate of the analysis, based on the mean depth of coverage. The recommended input amount for DNA (100 ng) was obtained for 72% of the samples. The two samples with input amounts <20 ng failed (mean coverages of 17× and 20×) but the 20 samples that had a starting amount between 35 ng and 95 ng (mean 58 ng ±17) yielded good sequencing libraries and sufficient coverage (Fig. S3). The mean coverages for most of these samples were in the same range (280× to 460×) as those with input amounts of 100 ng. Only two of these samples had a mean coverage of <250×. The recommended RNA input amount of 200 ng was reached for 54% of the samples. Also here, both samples with an input <20 ng failed the analysis while those with starting amounts >35 ng were successful.

## 4 Discussion

The TSO500 CGP kit has been on the market for more than 4 years now and has extensively been tested and validated for solid tumors [9, 10, 13, 14], but also for myeloid cancers [15, 16]. Of note, concordance of the OncoDEEP assay only states whether the results of both methods agree not taking into account any technological or operational differences. Both assays use probe capture‐based enrichment to interrogate >500 cancer‐related genes for the presence of small and large variations at the DNA level and the most clinically relevant genomic aberrations at the RNA level. Amplifications of the diagnostic‐relevant genes can be detected with both assays while gene deletions could only be analyzed with the OncoDEEP kit and thus could not be compared. The large size of the panels also allows to interrogate the samples for the pan‐cancer biomarkers MSI, TMB, and HRD.

Next to the differences in assay content, each test has its technical advantages and disadvantages. The recommended input amount of DNA and RNA at the time of the study was significantly lower for TSO500 (both 40 ng) compared to OncoDEEP (100 and 200 ng, respectively) but for the latter, these amounts have been adapted (Table 4). Drying of RNA in OncoDEEP requires an additional step in the protocol and might impact RNA quality. On the other hand, the ultrasound‐based shearing of DNA in the TSO500 assay needs a sonicator, which is more labor‐intensive compared to the enzymatic shearing in the OncoDEEP kit. Differences in pooling before the hybridization‐based capture and in the normalization of the final libraries will have an effect on time and cost efficiency. The simultaneous sequencing of a higher number of OncoDEEP libraries on the NextSeq550, compared to TSO500 libraries, is more economical. Additionally, since the HRD panel is an integral part of the OncoDEEP assay, no additional cost for a separate HRD panel is required although currently, only a very selective number of tumor types require this analysis [17]. Finally, OncoDEEP is an end‐to‐end solution resulting in final reports. Illumina connected insight (ICI) has now been released as a very similar add‐on decision support tool for TSO500 as well.

Currently, the number of interrogated genes for fusion detection of the OncoDEEP kit is largely insufficient. The 13 driver genes present in the current RNA kit will screen for important clinically actionable fusions but several others as well as new emerging diagnostic ones will be missed in specific tumor types. The TSO500 RNA panel analyzes fusions in 55 driver genes but this number can be significantly expanded by replacing it with the Illumina TruSight Pan‐cancer RNA panel that includes 507 driver genes for cancer‐related fusions. Moreover, the number of cycles in both PCR steps of the RNA library preparation of OncoDEEP differs from that of the DNA library preparation hampering the automation process to some extent. A different, much larger RNA panel is currently being tested as a working solution for OncoDEEP.

The comparative analysis of both assays was performed in seven diagnostic cancer centers in Belgium and the Netherlands. Importantly, since the same nucleic acid samples were used as starting material, differences in outcome are not expected to be due to the nucleic acid source. An exception was Center 5 (eight DNA samples) since DNA extracted by the Chelex method appeared to be incompatible with the starting material for OncoDEEP. This issue might be caused by the presence of PCR inhibitors or the higher proportion of denatured and shortened DNA derived by the Chelex protocol [18].

From the 674 variants (SNVs and indels) with VAF >5% found with TSO500, 68 (10.1%) were not detected with OncoDEEP. The reason for the majority of missed variants was due to the too low coverage of the total reads (52%) or variant reads (32%) at these positions based on the set thresholds in OncoKDM. The per center differences in the number of missed variants per analyzed sample indicate, however, that lab‐specific parameters could play a role. Our validation data showed a sensitivity of 90% and specificity of 100% for SNV detection (VAF >5%) for the OncoDEEP assay. Of note is that additional variants were detected in some of the 115 OncoDEEP‐specific genes but this analysis was outside the scope of this study. Moreover, the clinical relevance of drug dosing based on the presence of the pharmacogenomic‐relevant genes (*DPYD*, *UGT1A1*, *TPMT*, *CYP2D6*, etc.) has yet to be assessed [19]. Concordance of amplifications and rearrangements events were 100% and 94.4%, respectively, although the low number of fusion genes that are considered as drivers in the OncoDEEP panel significantly limits the comparison.

Analysis of the pan‐tumor biomarkers MSI and TMB also showed a very high concordance but required an assay‐specific TMB‐H threshold of 12 mut·Mb^−1^ for OncoDEEP and 16 mut·Mb^−1^ for TSO500 to obtain the highest agreement (96.6%). Discordant calls mostly had scores close to the threshold, which might advocate for an intermediate TMB class (TMB‐I). However, a more in‐depth comparison using additional samples in different tumor types is advised. TMB calculation clearly is panel‐dependent because of differences in gene content and size, mutation inclusion and exclusion criteria, and strategies for filtering out rare germline variants. Although there is no standard approach nor guideline for the TMB calculation, values of 10–16 have been described as cutoff [20, 21]. Finally, HRD scoring could not be sufficiently compared since only 22 samples of which four were references, could be compared. One study reported that among several HRD assays, the TSO500 data matched best with the Myriad results [22], not surprisingly since this assay assesses genomic instability with Myriad Genetics' proprietary algorithm. The OncoDEEP assay was not included in this study.

The OncoDEEP kit has not yet been reported in the literature for its diagnostic value. Our multicentric comparative analysis, however, demonstrated the weaknesses of this assay predominantly being the missing of about 10% of subtle variants and 6% of gene fusions, as well as the limited number of drivers for gene fusion detection. These data could have a significant effect on the patients' clinical outcome emphasizing the danger of using samples with poor QC values, which should be omitted. Therefore, we conducted an analytical validation of this novel CGP test by analyzing its performance characteristics for its potential diagnostic implementation. Intra‐ and inter‐run precision were maximal and a sensitivity and specificity of 90% and 100%, respectively, were obtained. The missed variants were mostly due to the too low coverage issue, which might require a lower number of OncoDEEP samples per sequencing run. The cost‐effectiveness of the OncoDEEP kit is achieved via the enzymatic DNA shearing included in the kit, the ability to pool eight libraries for hybridization, the single hybridization step, and its slightly shorter hands‐on time. The LoD experiments in which samples were mixed at variable contributions to the mix demonstrated that variants can be efficiently detected down to a threshold of VAF 5%. Due to this hard filter setting in OncoKDM, variants with VAF <5% could not be analyzed in this study. This shortcoming has now been dealt with in OncoKDM since the pathogenic variants with VAF between 1% and 5% will also be reported. The user can then accept or exclude these variants manually. A recent study using the OncoDEEP assay highlights the large impact of broad SNV and indel detection on treatment decisions for patients with advanced solid tumors, even without the inclusion of gene fusions and the pan‐cancer biomarkers [23]. The recently updated recommendations from ESMO advise the analysis of these biomarkers in patients with metastatic cancer where access to therapies is available [24, 25].

Despite the reported significant clinical value of large panel testing in solid tumors, the current lack of reimbursement in many countries significantly hinders its implementation in diagnostics. Due to the absence of standardized quality measures and published validation and utility data, labs may be inclined to substitute the cheaper small panel tests depriving patients to benefit from potentially life‐extending therapies. With this study, we aim to offer diagnostic laboratories general insight into the pros and cons of commercially available CGP assays, which soon will enter most cancer hospitals. In summary, with an updated RNA panel, the OncoDEEP assay can efficiently be used for comprehensive tumor profiling to detect clinically actionable gene alterations and biomarkers for improved patient management and its subsequent clinical benefit.

## 5 Conclusion

Comprehensive genomic profiling is going to replace the small NGS panels in molecular cancer diagnostics since it provides clinically relevant information on all somatic variants as well as genomic biomarkers with clinical value. The choice of the CGP method for diagnostic implementation, however, has to be critically considered based on the requirements of the laboratory. Here, we compared two CGP assays and discussed the pros and cons of both. In addition, future improvements should also be taken into account in this fast‐evolving field.

## Conflict of interest

The authors have no conflict of interest to declare. The company OncoDNA provided the OncoDEEP kits as well as the data analyses in OncoKDM for free.

## Author contributions

GF, EJMS, WFJvI, and BM conceived and coordinated the study. JvdM, JVH, GB, WWJdL, HJD, EJMS, and BM provided the study material. GF, EG, SB, ADC, SV, MdB, AMLJ, ID, ZO, and WFJvI collected and assembled the data. GF and PJV performed data analysis and interpretation. GF wrote the manuscript. All authors read, reviewed, and approved the manuscript.

## Peer review

The peer review history for this article is available at https://www.webofscience.com/api/gateway/wos/peer‐review/10.1002/1878‐0261.13812.

## Supporting information


**Fig. S1.** Percentages of missed variants (Y axis) due to a too low mean coverage (<250 reads; blue bars) or too low uniformity of coverage (<90%; red bars) of the samples carrying these missed variants, provided per center.
**Fig. S2.** Limit‐of‐detection (LoD) for variant calling tested by mixing two samples at different ratios (75%, 25%, and 10%).
**Fig. S3.** Plot of the mean coverage obtained for the 22 retrospective diagnostic samples with a DNA input amount < 100 ng.
**Table S1.** Number (*n*) of samples included per tumor type.

**Table S2.** Gene content of the TSO500 and OncoDEEP assays for analysis of SNVs–indels, gene fusions, splice variants, and amplifications.
**Table S3.** Pathogenic and likely pathogenic variants that were absent in the OncoDEEP analysis, and the reason why.
**Table S4.** Amplified genes with their fold change (FC) in TSO500 and copy number (CN) in OncoDEEP.
**Table S5.** Comparison of the presence of gene fusions and exon skipping events in the TSO500 and OncoDEEP data.
**Table S6.** Comparison of HRD scoring on 10 diagnostic samples with the OncoDEEP and TSO500 CGP panels.

## Data Availability

Data sharing of the diagnostic samples is not feasible because of ethical constraints. Upon request, parts of the fully anonymized data can be provided. The additional supporting information can be found online, as part of this paper.
