# Supplementary material for: Analysis of comprehensive genomic profiling of solid tumors with a novel assay for broad analysis in clinical diagnostics
Source: Mol Oncol. 2025 Jan 31;19(6):1797–810. doi: 10.1002/1878-0261.13812 (PMC12161465; doi:10.1002/1878-0261.13812)
Supplement: Supplementary file 1 — Fig. S1. Percentages of missed variants (Y axis) due to a too low mean coverage (<250 reads; blue bars) or too low uniformity of coverage (<90%; red bars) of the samples carrying these missed variants, provided per center. Fig. S2. Limit‐of‐detection (LoD) for variant calling tested by mixing two samples at different ratios (75%, 25%, and 10%). Fig. S3. Plot of the mean coverage obtained for the 22 retrospective diagnostic samples with a DNA input amount < 100 ng. Table S1. Number (n) of samples included per tumor type. [file MOL2-19-1797-s002.docx]

**Supporting information**

**Supporting tables**

**TABLE S1.** Number (n) of samples included per tumor type. We included 234 unique tumor samples across >20 tumor types. The ‘Other’ category represents tumor types with less than 3 samples and include adrenal, jejunum, mesothelioma, neuro-endocrine, salivary, stomach, testis and thyroid.

**TABLE S2.** Gene content of the TSO500 and OncoDEEP assays for analysis of SNVs-indels, gene fusions, splice variants and amplifications.

*Separate excel table due to its large content and different tabs.*

**TABLE S3.** Pathogenic and Likely Pathogenic variants that were absent in the OncoDEEP analysis, and the reason why. ID: the number of the missed variant of that center (C1 to C7).

**TABLE S4**. Amplified genes with their fold change (FC) in TSO500 and copy number (CN) in OncoDEEP; nd: not detected.

**TABLE S5.** Comparison of the presence of gene fusions and exon skipping events in the TSO500 and OncoDEEP data.

**TABLE S6.** Comparison of HRD scoring on 10 diagnostic samples with the OncoDEEP and TSO500 CGP panels. HRD positivity (Pos) is due to the GIS score ≥ the threshold (Thr) and/or the presence of a pathogenic BRCA variant.

**Supporting Figures**

**Figure S1**

**
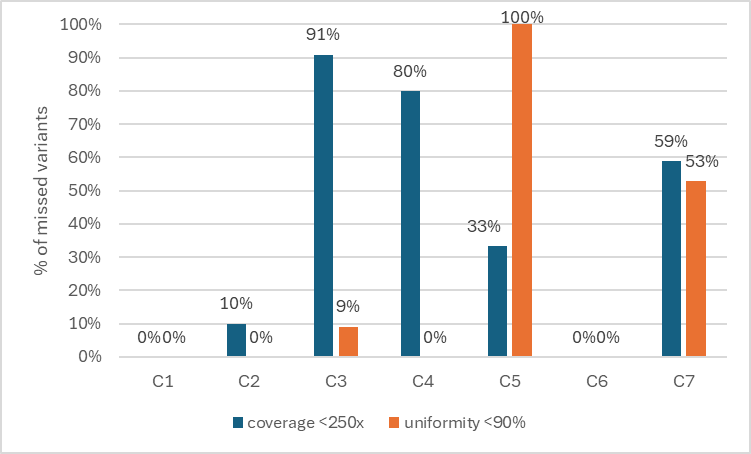
**

**FIG S1.** Percentages of missed variants (Y axis) due to a too low mean coverage (<250 reads; blue bars) or too low uniformity of coverage (<90%; red bars) of the samples carrying these missed variants, provided per center.

**Figure S2**

**
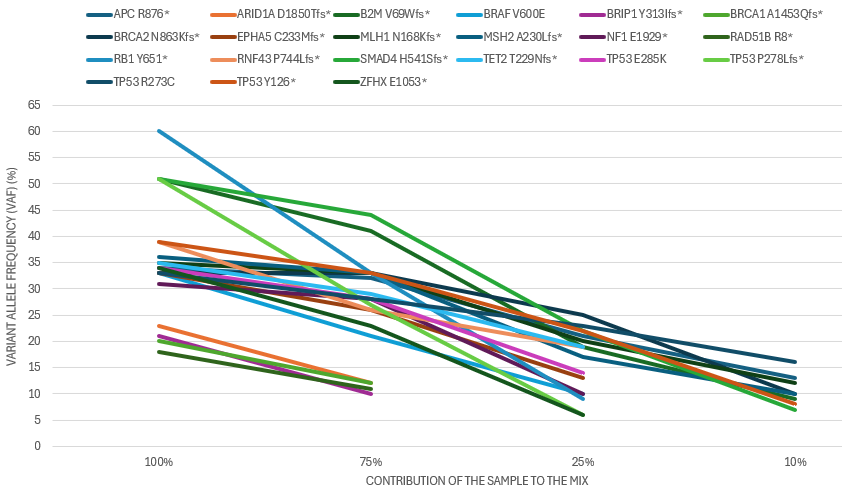
**

**FIG S2.** Limit-of-detection (LoD) for variant calling tested by mixing 2 samples at different ratio’s (75%, 25% and 10%). The undiluted samples indicate the 100% contribution. VAF detection threshold was set at 5%.

**Figure S3**

**
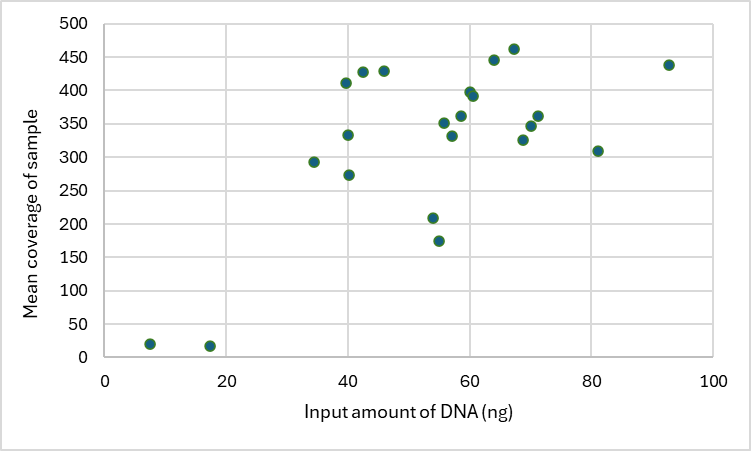
**

**FIG S3.** Plot of the mean coverage obtained for the 22 retrospective diagnostic samples with a DNA input amount <100 ng.
